# Neurodegeneration, memory loss, and dementia: the impact of biological clocks and circadian rhythm

**DOI:** 10.52586/4971

**Published:** 2021-09-30

**Authors:** Kenneth Maiese

**Affiliations:** 1 Cellular and Molecular Signaling, New York, NY 10022, USA

**Keywords:** Alzheimer’s disease, Autophagy, Circadian rhythm, Dementia, Erythropoietin, Forkhead, FoxO, Mechanistic target of rapamycin (mTOR), Parkinson’s disease, Silent mating type information regulation 2 homolog 1, *wingless*, Wnt

## Abstract

**Introduction:**

Dementia and cognitive loss impact a significant proportion of the global population and present almost insurmountable challenges for treatment since they stem from multifactorial etiologies. Innovative avenues for treatment are highly warranted.

**Methods and results:**

Novel work with biological clock genes that oversee circadian rhythm may meet this critical need by focusing upon the pathways of the mechanistic target of rapamycin (mTOR), the silent mating type information regulation 2 homolog 1 (*Saccharomyces cerevisiae*) (SIRT1), mammalian forkhead transcription factors (FoxOs), the growth factor erythropoietin (EPO), and the *wingless* Wnt pathway. These pathways are complex in nature, intimately associated with autophagy that can maintain circadian rhythm, and have an intricate relationship that can lead to beneficial outcomes that may offer neuroprotection, metabolic homeostasis, and prevention of cognitive loss. However, biological clocks and alterations in circadian rhythm also have the potential to lead to devastating effects involving tumorigenesis in conjunction with pathways involving Wnt that oversee angiogenesis and stem cell proliferation.

**Conclusions:**

Current work with biological clocks and circadian rhythm pathways provide exciting possibilities for the treating dementia and cognitive loss, but also provide powerful arguments to further comprehend the intimate and complex relationship among these pathways to fully potentiate desired clinical outcomes.

## 2. Introduction

Neurodegenerative disorders pose a significant challenge for diagnosis, preventing disease progression, and providing treatment. Cognitive loss in relation to Alzheimer’s disease (AD) is an excellent example since diseases that include AD are the result of multiple underlying mechanisms [1–6] (Table 1). For example, many pathways may lead to memory loss and involve neuronal and vascular cell injury related to metabotropic receptors, lipid dysfunction, cellular metabolic dysfunction with diabetes mellitus (DM), astrocytic cell injury, *β*-amyloid (A*β*), heavy metal disease, loss of access to bright light, tau, mitochondrial damage, oxidative stress, acetylcholine loss, and excitotoxicity [1, 3, 6–30].

In addition, cognitive disorders raise significant financial concerns [1, 31–34]. Greater than 800 billion United States dollars (USD) per year are required to treat dementia equaling approximately 2 percent of the global Gross Domestic Product. Social and medical services by the year 2030 may possibly equal 2 trillion USD per year in the United States. Currently, greater than 5 million patients have AD and it is estimated that 4 million receive care at a yearly cost of 3.8 billion USD. Furthermore, the market revenue to provide treatments for AD may not be fully appreciated, but at minimum it may be greater than 11 billion USD. Many new social and medical services will be necessary to meet this challenge such that 60 million additional care workers will be needed [35–37]. These projections do not consider that all cases of dementia may not have been identified and diagnosed at this time [38, 39].

Cognitive loss impacts a large spectrum of the population. Dementia in the United States affects greater than 5 million people [4]. Many of these cases, 60 percent, are diagnosed as AD [4, 6, 17, 40–43]. Case of AD that are familial in origin comprise under 2% of all cases [4]. In familial AD that affects 200 families worldwide, mutations in the presenilin 1 or 2 genes occurs and an autosomal dominant mutated amyloid precursor protein (APP) gene exists. In these familial AD patients, illness can present prior to 55 years of age [44–46]. Familial AD can be the result of mutations in chromosome 21 leading to changes in APP, mutations in chromosome 14 causing changes in presenilin 1, and mutations in chromosomes 1, 14, and 21 such that mutations in chromosome 1 lead to changes in presenilin However, it is the sporadic version of AD that leads to illness in patients over age 65 and represents the cases of AD in ten percent of the population in the world. The *ϵ*4 allele of the apolipoprotein E (APOE) gene represents an additional risk of developing AD in the sporadic group.

## 3. Biological clocks and circadian rhythm pathways for dementia treatment

**Fig. 1.**
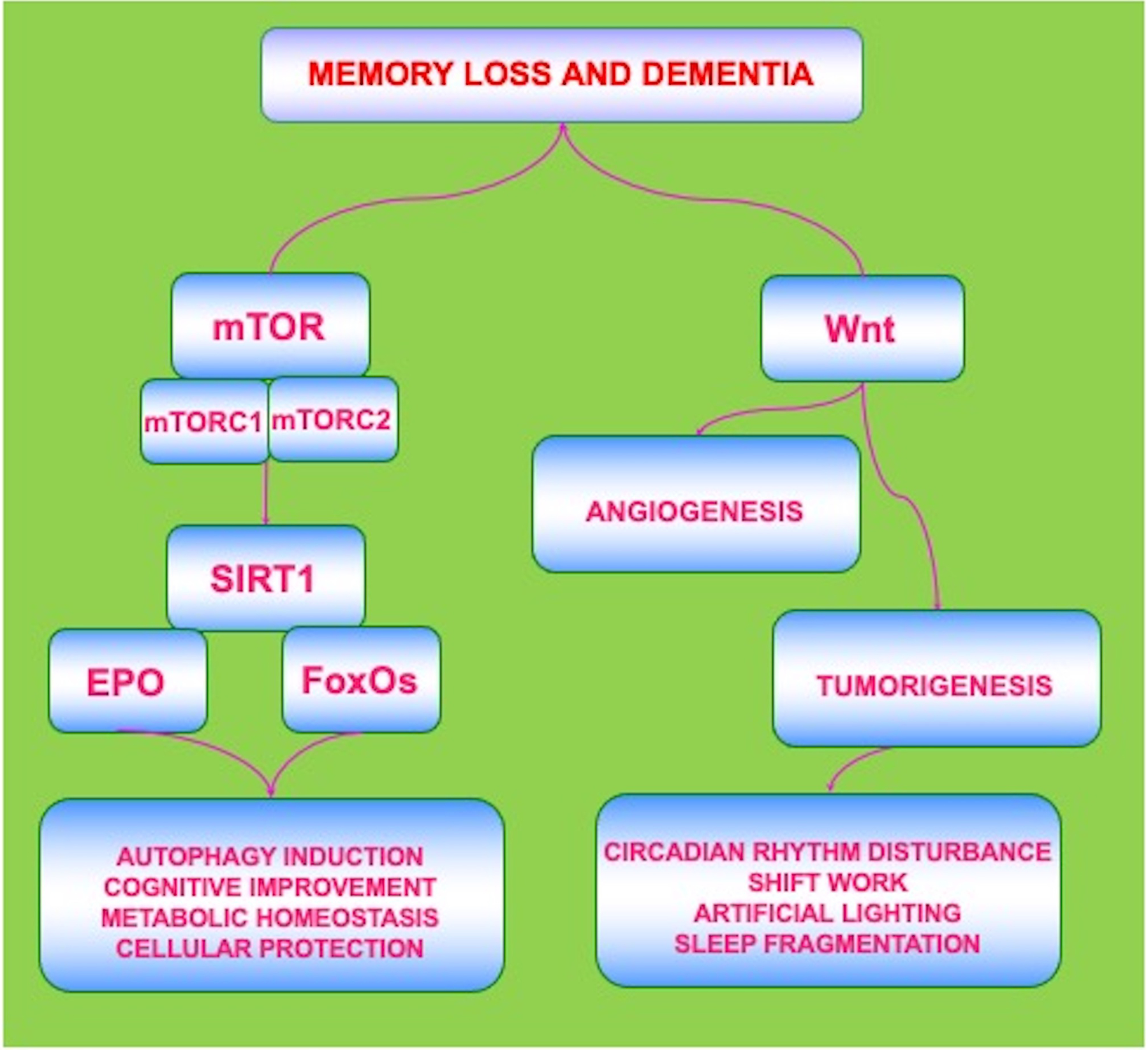
Biological Clock Pathways Are Complex and May Yield Variable Outcomes. The circadian biological clock gene pathways are intricately related but complex in nature. The pathways of the mechanistic target of rapamycin (mTOR), the silent mating type information regulation 2 homolog 1 (*Saccharomyces cerevisiae*) (SIRT1), mammalian forkhead transcription factors (FoxOs), the growth factor erythropoietin (EPO), and the *wingless* Wnt/*β*-catenin pathway can lead to beneficial outcomes and employ autophagy induction that may provide cellular protection, metabolic homeostasis, and prevent dementia and cognitive loss. Yet, biological clocks and alterations in circadian rhythm, such as during sleep disruption and fragmentation, also have the potential to lead to devastating effects involving tumorigenesis in conjunction with pathways involving Wnt that oversee angiogenesis and stem cell proliferation. Circadian rhythm disruption can result from shift work, exposure to artificial lighting, and from sleep fragmentation. A fine balance in the oversight of circadian biological clock gene pathways is required to foster safe and efficacious clinical outcomes for the treatment of dementia and cognitive loss.

Current attempts to treat dementia such as with cholinesterase inhibitors may lead to a decrease in the presenting symptoms but ultimately do not block the progression of the disease, such as in AD [27, 45, 47, 48]. Other treatments for cognitive loss can focus on metabolic disorders, such as diabetes mellitus (DM) [1, 20, 27, 41, 49, 50], and on vascular disease [19, 45, 51–53]. Yet, there exist other risks for developing vascular cognitive loss that can affect the efficacy of treatments such as tobacco use, alcohol consumption, hypertension, and a low level of education [20, 39, 54–57]. With reference to metabolic disease, tight glucose control in the serum in combination with early diagnosis of DM may assist to limit the progression of the disease, but complications from DM can still ensue [6, 58–69]. Given the need for novel strategies directed against memory loss and dementia, exciting new avenues of development are now focusing upon biological clock mechanisms and include the pathways of the mechanistic target of rapamycin (mTOR), its associated pathways of mTOR Complex 1 (mTORC1), mTOR Complex 2 (mTORC2), the silent mating type information regulation 2 homolog 1 (*Saccharomyces cerevisiae*) (SIRT1), mammalian forkhead transcription factors (FoxOs), the growth factor erythropoietin (EPO), and the *wingless* pathway of Wnt pathway (Fig. 1).

Biological clocks and circadian rhythm pathways are vital components in the onset of nervous system disorders, memory loss, and dementia [6, 34, 39, 70–76] (Table 1). Changes in the function of biological clock pathways can impact cellular metabolic homeostasis [6, 76–85], cancer [6, 80, 81, 84, 86–89], energy metabolism and aging [70, 74, 77, 84, 90], mitochondrial energy maintenance [76, 81, 91, 92], renal disease [78, 86], and viral diseases [72, 93–101]. Circadian rhythm in mammals is controlled in a region over the optic chiasm that detects light with retinal photosensitive ganglion cells in the suprachiasmatic nucleus (SCN) [6, 84, 98]. With the exposure to external light, biological clock genes oversee biochemical cell transmissions, physiological process in the body, and changes in behavior. The SCN controls the temperature of the body, cortisol and melatonin release, and oxidative stress responses through a connected system among the hypothalamic nuclei, pineal gland, and vasoactive intestinal peptide [88, 102, 103]. As part of the biological clock gene group, members of the basic helix-loop-helix-PAS (Period-Arnt-Single-minded) transcription factor family, that include CLOCK and BMAL1 [104], control gene expression of *Cryptochrome* (*Cry1 and Cry2*) and *Period* (*Per1, Per2, and Per3*) [6, 78, 84, 86, 105–107]. Modulation of these pathways and auto-feedback interactions are controlled by PER:CRY heterodimers that block transcription during nuclear translocation promoted by CLOCK:BMAL1 complexes. Other regulatory pathways that can be activated by CLOCK:BMAL1 heterodimers include ROR*α* and retinoic acid-related orphan nuclear receptors REV-ERB*α*, also termed NR1D1 (nuclear receptor subfamily 1, group D, member 1). The REV-ERB*α* and ROR*α* receptors attach to retinoic acid-related orphan receptor response elements (ROREs) that exist in the BMAL1 promoter to block and promote rhythmic transcription of BMAL1 by RORs and REV-ERBs, respectively. REV-ERBs can inhibit transcription to lead to circadian oscillation of BMAL1 [74, 105].

With neurodegeneration and aging studies, experimental studies with Parkinson’s disease (PD) using 6-hydroxydopamine (6-OHDA) during chronic treatment with levodopa show depressed levels of BMAL1 and ROR*α*, indicating that memory loss in PD patients also may be a result of medication that alters circadian rhythm clock genes [106]. Cognitive impairment with memory loss and neuronal injury may occur as a result of sleep fragmentation during extended space flight which alters circadian rhythm [108, 109]. Changes in the DNA methylation of biological clock genes may foster memory loss and changes in behavior since rhythmic methylation of BMAL1 has been shown in the brains of individuals with AD [70]. In experimental studies AD using mice, significant alterations have been observed in RNA clock gene expression that may suggest a dysfunction in the clock pathways during cognitive loss [110].

## 4. Circadian rhythm disruption and the *wingless* wnt pathway

Lifespan can be affected by biological clock genes. Lifespan in *Drosophila melanogaster* is decreased through three arrhythmic mutants involving ClkAR, cyc0 and tim0. In addition, mutations in ClkAR with increasing age can result in dysfunction with ambulation. Through the promotion of Clk function, the locomotor deficits in *Drosophila* were reversed. This loss of function appears linked to the absence of dopaminergic neurons instead of insults from oxidative stress [75]. Other studies in *Drosophila* also suggest negative effects with alterations in circadian rhythm [6, 80, 84] (Table 1). For example, TIMELESS, a mammalian homolog of *Drosophila* circadian rhythm gene, can lead to cell death and has increased expression in nasopharyngeal carcinoma. During increased TIMELESS expression, cell growth pathways are fostered that involve the *wingless* pathway of Wnt/*β*-catenin and resistance against chemotherapy to lead to cell apoptosis, such as with cisplatin, is increased [89]. Wnt proteins are cysteine-rich glycosylated proteins that can affect development of neurons, immune system function, tissue fibrosis, angiogenesis, stem cell development, and cancer [111–114]. Yet, detrimental effects with Wnt pathways can result to promote increased vascular growth of tumors [111, 115, 116] and tumorigenesis [40, 117–121]. As a result, these mechanisms may work in conjunction with TIMELESS. There also is evidence for sleep fragmentation and disruption of biological clock genes with shift work to indicate that these lighting and international travel are other examples that can lead to circadian rhythm disturbance [79]. Sleep deprivation affects circadian rhythm and can prevent the clearance of A*β*, *α*-synuclein, and tau that are tied to the progression of nervous system disorders that include AD and PD [34, 109]. Some work suggests that female healthcare workers with extended night shift work may be at enhanced risk for breast cancer [122]. The circadian gene *hClock* during increased expression also can lead to cancer and colorectal cancer metastatic disease through promotion genes that activate angiogenesis [123].

## 5. The mechanistic target of rapamycin (mTOR) and autophagy

Circadian clock genes rely upon pathways of both autophagy and the mechanistic target of rapamycin (mTOR) [6, 84, 124–126] (Table 1). Circadian rhythm dysfunction can lead to changes in the induction of autophagy especially during cognitive loss [72, 81, 84, 92, 127–129]. Autophagy plays a vital role in multiple diseases of the nervous system and can sequester and remove intracellular deposits during AD [19, 41, 130, 131], amyotrophic lateral sclerosis [48, 132, 133], Huntington’s disease (HD) [19, 134], traumatic brain injury [135–137], and PD [83, 130, 135, 138–140]. This removal of toxic intracellular substances may be important to maintain memory and cognition. As part of a programmed cell death pathway, autophagy is tied to oxidative stress [2, 29, 66, 67, 71, 141–145]. Autophagy pathways can recycle cytoplasmic organelles and components for tissue remodeling [19, 146] and can eliminate non-functional organelles [6, 71, 142, 147]. Macroautophagy reuses organelles in cells and packages cytoplasmic proteins into cellular components termed autophagosomes. Once associated with lysosomes, the autophagosomes are degraded to begin another process for the recycling of organelles [19]. Microautophagy promotes invagination of lysosomal membranes to allow for the digestion of cell cytoplasm components. Chaperone-mediated autophagy employs cytosolic chaperones to transport cytoplasmic cell components across lysosomal membranes.

Previous studies also suggest in experimental studies with AD that a baseline cyclic circadian rhythm that controls autophagy is necessary to reduce A*β* deposition and prevent memory loss [129, 148]. Alterations in environmental homeostasis [82, 129, 149] can alter circadian rhythm that results in loss of cognitive ability [2, 19, 49, 50, 84, 150]. Sleep fragmentation also can produce changes in hippocampal autophagy proteins and decrease memory function [4, 127, 151–154]. Cellular protection is dependent on the activation of autophagy with circadian clock proteins during insults with stroke, since loss in the function of the PER1 circadian clock protein can increase cerebral ischemia [128].

In regard to the mTOR pathway, mTOR is a 289-kDa serine/threonine protein kinase and is vital during nervous system disease and memory loss [2, 19, 20, 25, 49, 155–157]. mTOR is also known as the mammalian target of rapamycin and the FK506-binding protein 12-rapamycin complex-associated protein 1 [19, 85, 158, 159]. mTOR is the main component of the protein complexes mTOR Complex 1 (mTORC1) and mTOR Complex 2 (mTORC2) [160–162]. mTORC1 and mTORC2 are then divided into additional components [2, 107, 163–165]. mTORC1 is composed of Raptor, Deptor (DEP domain-containing mTOR interacting protein), the proline rich Akt substrate 40 kDa (PRAS40), and mammalian lethal with Sec13 protein 8, termed mLST8 (mLST8) [20, 40, 166]. mTORC1 activity is controlled through a number of pathways that includes PRAS40 by blocking the association of p70 ribosomal S6 kinase (p70S6K) and the eukaryotic initiation factor 4E (eIF4E)-binding protein 1 (4EBP1) with Raptor [167, 168]. Rapamycin is an agent that can inhibit mTOR activity [164, 169–172]. Rapamycin blocks the activity of mTORC1 through its association with immunophilin FK-506-binding protein 12 (FKBP12) that attaches to the FKBP12 -rapamycin-binding domain (FRB) at the carboxy (C) -terminal of mTOR to impede the FRB domain of mTORC1 [4]. mTORC2 is composed of Rictor, Deptor, mLST8, the mammalian stress-activated protein kinase interacting protein (mSIN1), and the protein observed with Rictor-1 (Protor-1) [167, 173, 174]. mTORC2 oversees remodeling of the cytoskeleton through PKC*α* and the migration of cells through the Rac guanine nucleotide exchange factors P-Rex1 and P-Rex2 and through Rho signaling [175]. Cognitive decline can be associated with the loss of mTOR activity and altered circadian rhythm during extended space flight [108]. Ischemia in the brain that leads to stroke may be altered by alteration in circadian rhythm genes and fluctuations in the activity of mTOR [124, 128]. Other studies suggest that the absence of period2 (PER2), a mammalian circadian clock protein, can increase mTOR activity and chemotherapy drug resistance [125].

mTOR also maintains a relationship with the silent mating type information regulation 2 homolog 1 (*Saccharomyces cerevisiae*) (SIRT1). SIRT1 maintains an inverse relationship with mTOR [19, 176–180]. SIRT1 can also affect pathways of autophagy [49, 65, 163, 178, 181–186]. SIRT1 activity can lead to the expansion of neurites and promote the survival of neurons during conditions that limit nutrients that involves mTOR inhibition [187]. SIRT1 can foster growth of tumors during autophagy induction that requires the blockade of mTOR, indicating that autophagy and SIRT1 can be targeted to control tumorigenesis [183]. SIRT1 is necessary to foster autophagy and mTOR inhibition during oxidative stress to preserve mitochondrial function in embryonic stem cells [188]. During periods of elevated serum glucose, SIRT1 can block mTOR to offer vascular cell protection [189]. SIRT1 with the blockade of mTOR activity can increase photoreceptor cell survival [177] and limit cell senescence [190]. It is also important to note that some pathways that lead to nerve cell injury require a relationship between mTOR and SIRT1 that is symbiotic. During the loss of dopaminergic neuronal cells, it has been observed that a balance in activities of SIRT1, mTOR, and forkhead transcription factors are required to promote neuronal cell survival [191]. It also has been demonstrated that SIRT1 and mTOR absence during obesity can suppress core circadian components CLOCK and BMAL1 and lead to loss of metabolic cellular homeostasis. The agent metformin, an inhibitor of mTOR activity [4, 65, 72], can prevent such processes during obesity in experimental mouse models and can reverse the loss of SIRT1 function during inhibition of the circadian components CLOCK and BMAL1 [192].

## 6. The silent mating type information regulation 2 homolog 1 (Saccharomyces cerevisiae) (SIRT1)

Biological clock pathways closely rely upon SIRT1 [6, 84, 85, 91, 193, 194] (Table 1). SIRT1 is a histone deacetylase that can transfer acetyl groups from *ϵ*-*N*-acetyl lysine amino acids to the histones of deoxyribonucleic acid (DNA) to control transcription [19, 45, 48, 84, 85, 152, 195–200]. As noted above, SIRT1 plays a critical role in nervous system diseases [164, 199, 201, 202] that also are dependent upon autophagy regulation [113, 178, 185, 186, 190, 203]. Other work focuses on SIRT1 to control the expression of clock genes through PER2 deacetylation [204]. SIRT1 its ability to control multiple biological clock gene pathways indicates that loss of SIRT can impact circadian rhythm cycles and result in memory loss and AD [110].

Through SIRT1 pathways, the coenzyme ß-nicotinamide adenine dinucleotide (NAD^+^) has an important function with clock genes that is linked to mTOR [20, 66, 72, 192, 205]. Control of circadian rhythm by SIRT1 and melatonin can impact glucose tolerance in cells [102]. Dementia onset can be dependent upon melatonin, a pineal hormone that controls circadian rhythm [81, 88, 95], as well as mTOR through autophagy induction [90, 206]. During the process of aging, circadian rhythm cycles involving melatonin can affect infection with coronavirus disease of 2019 (COVID-19) [94], cellular metabolism [90, 103], mitochondrial dysfunction [81], oxidative stress [207, 208], and inflammatory mediators [206, 209]. In addition, SIRT1 can affect biological clock rhythm through stem cell function [210] and inflammation during obesity [91] and neurodegeneration [209]. Cellular NAD^+^ pools fluctuate with circadian rhythmicity and with aging [72]. SIRT1 in connection with CLOCK:BMAL1 can control the circadian expression of nicotinamide phosphoribosyltransferase (NAMPT) that is required for NAD^+^ production. SIRT1 also through the NAMPT promoter can promote the circadian synthesis of its own coenzyme [211]. Yet, NAD^+^ cellular pools can become depleted during impairment of mitochondrial function to result in cell injury with cellular NAD^+^ pools oscillating with free nicotinamide levels and promoting cell injury, metabolic dysfunction, and loss of cognitive function [205].

SIRT1 regulation of biological clock genes also can affect cognitive function though growth factors, such as EPO [161, 197, 212–214]. The *EPO* gene is present on chromosome 7 and represents a single copy in a 5.4 kb region of the genomic DNA [215, 216]. The gene encodes for a polypeptide chain protein that has 193 amino acids [64, 217]. EPO later undergoes the removal of a carboxy-terminal arginine^166^ in the mature human and recombinant human EPO (rhEPO). A protein with a molecular weight of 30.4 kDa and 165 amino acids is generated as the mature protein [218–221]. EPO expression occurs in the brain, uterus, and liver [64, 161, 164, 215, 216, 222, 223], but the principal site for the production and secretion of EPO is the peritubular interstitial cells of the kidney [216, 217, 224–227]. It is important to note that expression of EPO is overseen by oxygen tension changes and not by the concentration of red blood cells [64, 228, 229].

In relation to SIRT1, EPO prevents metabolic dysfunction by modulating adipose energy homeostasis in adipocytes through the combined activation of peroxisome proliferator-activated receptor-*α* (PPAR-*α*) and SIRT1 [213] (Table 1). EPO promotes vascular cell protection in the brain through SIRT1 nuclear subcellular trafficking and blocks mitochondrial depolarization, cytochrome c release, BCL2 associated agonist of cell death (Bad) activity, and caspase activation [212]. EPO can increase human cardiomyocyte survival through SIRT1 activation during chemotherapy toxicity [197] and prevent brain neuronal cell loss through the up-regulation of SIRT1 [214]. EPO can block memory loss during AD [5, 43], control metabolic pathways [230, 231], and block mitochondrial dysfunction [197, 216, 222, 232–234]. However, control of biological clock gene pathways appear to be necessary for EPO and SIRT1 to offer cellular protection. Some studies indicate that during hypoxia specific clock genes, that include *BMAL1* and *PER2*, are required for the production of EPO [235].

EPO also relies upon mTOR to affect cellular survival. EPO employs mTOR to foster neuronal regeneration through autophagy and apoptotic pathways [20, 203, 236–239]. EPO prevents apoptosis during A*β* exposure with mTOR activation to prevent caspase activation [240]. EPO can increase the survival of microglia during oxidative stress through mTOR signaling pathways [241]. EPO oversees mTOR, protein kinase B (Akt) [232, 242, 243], and proline rich Akt substrate 40 kDa (PRAS40) to promote the survival of neurons during oxygen-glucose deprivation [244].

## 7. Mammalian forkhead transcription factors (FoxOs)

Mammalian FOXO proteins of the O class are transcription factors and play a significant role in the nervous system. FoxO family members include FOXO1, FOXO3, FOXO4, and FOXO6 [67, 164, 245–247] (Table 1). FoxO proteins bind to deoxyribonucleic acid (DNA) through the FoxO-recognized element in the *C-*terminal basic region of the forkhead DNA binding domain. With the binding to DNA by FoxOs, target gene expression is blocked or promoted through fourteen protein-DNA contacts with the primary recognition site located at *α*-helix H3. Phosphorylation or acetylation of FoxOs can change the binding of the *C-*terminal basic region to DNA to inhibit FoxO transcriptional activity [48, 152, 199, 248]. FoxOs are intimately connected circadian rhythm since they are linked to SIRT1 [4, 34, 48, 85, 248–252]. For example, insulin-phosphatidylinositol 3-kinase (PI3K) signaling that occurs in the liver is overseen by FoxO3 control of circadian rhythmicity through modulation of Clock. Loss of FoxO3 impairs the circadian amplitude and rhythmicity [253]. Autophagy induction also is dependent on mammalian FOXO proteins of the O class [12, 164, 202, 254, 255]. FoxO1 transcription factors [256] oversee the myelination of nerves that requires oligodendrocyte progenitor cells and determine the progression of disorders that include multiple sclerosis [257]. Additional studies indicate that epigenetic changes in DNA methylation and genetic variations of FoxO3a and FoxO1 also can affect demyelinating disorders [258]. Yet, it is important to state that a fine balance in FoxO activity is necessary to lead to the protection of cells since activation of FoxOs with autophagy can be beneficial. Sequestering and clearance of detrimental intracellular accumulations by FoxOs and autophagy can lead to increased survival of neurons [246, 259, 260].

In regard to SIRT1, blockade of the activity of FoxOs by SIRT1 can promote cell survival [19, 67, 249–251]. However, FoxOs can attach to the SIRT1 promoter region to further change forkhead transcription [181]. This mechanism permits FoxOs to use auto-feedback mechanisms to regulate the activity of SIRT1. FoxO proteins, including FoxO1, can oversee SIRT1 transcription and increase the expression of SIRT1 [261]. These studies suggest an intimate relationship between SIRT1 and FoxOs. Interestingly, SIRT1 and FoxOs can synergistically increase cell survival. SIRT1 and FoxO3a can work in unison to block memory loss and A*β* brain toxicity, mitochondrial dysfunction, and oxidative stress [5, 152, 262, 263].

## 8. Future perspectives

Neurodegenerative disorders that involve cognitive loss and dementia impact a significant proportion of the world’s population and lead to a large financial burden for all nations. Adding to these concerns is the knowledge that cognitive disorders present almost insurmountable challenges for treatment since they are multifactorial in origin and can result from multiple pathways that involve A*β*, tau, metabotropic receptors, excitotoxicity, lipid dysfunction, mitochondrial damage, astrocyte injury, loss of access to bright light, heavy metal disease, acetylcholine loss, oxidative stress, and metabolic dysfunction that involves DM. Novel new therapeutic strategies are desperately warranted. New investigations may meet this need with work that highlights biological clock genes that oversee circadian rhythm and involve the pathways of mTOR, SIRT1, FoxOs, EPO, and the Wnt/*β*-catenin pathway (Fig. 1). These pathways are complex in nature and intimately tied to autophagy induction that can sequester intracellular accumulations and potentially reduce cognitive loss under some conditions. Dysfunctional changes in biological clock genes and circadian rhythm can result in motor deficits, memory impairment, and the progression of dementia. Even chronic treatment regimens that occur during PD can alter circadian rhythm function and foster dementia. The pathways of autophagy may be one mechanism to oversee circadian rhythm homeostasis that can become lost during conditions of chronic sleep fragmentation.

The pathways that impact circadian rhythm have an intricate relationship that can lead to both beneficial as well as detrimental clinical effects. For example, blockade of mTOR activity can change circadian rhythm, affect memory function, and increase neuronal cell injury such as during stroke. SIRT1 can oversee the production of NAD^+^ pools that have been tied to circadian rhythmicity and if these cellular pools become depleted, cell injury and metabolic dysfunction can ensue with cognitive loss. Furthermore, without circadian rhythm control, the protective capability of EPO and SIRT1 may become absent and lead to mitochondrial dysfunction and the loss of cognition. In regard to FoxOs, SIRT1 and FoxOs may be required to work in unison to limit cognitive loss, mitochondrial dysfunction, and oxidative stress. Yet, it is important to remember that Wnt pathways that function in conjunction with circadian clock gene pathways, such as TIMELESS, may promote new angiogenesis and tumorigenesis. In addition, other circadian genes that include *hClock* also may promote metastatic colorectal cancer through the promotion of angiogenesis-related gene activity and vascular cell growth.

These observations serve to form a strong foundation for the further investigation of biological clock genes and circadian rhythm in regards to their significant role in neurodegenerative disorders such as dementia. The circadian pathways involving mTOR, SIRT1, FoxOs, EPO, and the Wnt can offer considerable potential for the understanding and treatment of memory loss and neurodegnerative disorders. Yet, it is the intimate and complex relationship among these pathways that is most intriguing and potentially offers the greatest insight to harness this knowledge for the innovative treatment of dementia.

## Figures and Tables

**Table 1:** Highlights

Neurodegeneration and Dementia: Circadian Rhythm Biological Clock Gene Pathways
• Cognitive loss in relation to Alzheimer’s disease is an excellent example of complex disorders that are multi-factorial in origin and may involve several mechanisms as etiologies that include cellular injury from β-amyloid, tau, metabotropic receptors, excitotoxicity, lipid dysfunction, mitochondrial damage, loss of access to bright light, acetylcholine loss, oxidative stress, and metabolic dysfunction with diabetes mellitus.
• Current strategies to treat cognitive loss are limited and do not adequately address disease onset and progression. Innovative work with biological clock genes that oversee circadian rhythm can offer new strategies for the treatment of dementia that employ the pathways of the mechanistic target of rapamycin (mTOR), the silent mating type information regulation 2 homolog 1 *(Saccharomyces cerevisiae*) (SIRT1), mammalian forkhead transcription factors (FoxOs), the growth factor erythropoietin (EPO), and the *wingless* Wnt/β-catenin pathway.
• Autophagy in combination with biological clock gene pathways are dependent upon mTOR. Studies suggest that a basal circadian rhythm that modulates autophagy and mTOR pathways involving mTOR Complex 1 (mTORC1) and mTOR Complex 2 (mTORC2) may be necessary to prevent cognitive decline and cellular toxicity with amyloid deposition. mTOR also holds an inverse relationship with SIRT1 and these pathways may be necessary to support core circadian components CLOCK and BMAL1 and prevent cellular metabolic dysfunction.
• SIRT1,a histone deacetylase, regulates ß-nicotinamide adenine dinucleotide (NAD^+^) cellular NAD^+^ pools that fluctuate with circadian rhythmicity and can impact cell function, metabolism, and loss of cognitive function. Oversight with SIRT1 of circadian rhythm pathways may be required for growth factor EPO cellular production and protection.
• FoxOs that can control circadian rhythmicity, such as through the modulation of Clock, can also bind to SIRT1 promoter regions to function through autofeedback mechanisms to regulate SIRT1 activity. SIRT1 and FoxOs can work in unison to block cognitive loss and prevent amyloid toxicity, mitochondrial dysfunction, and oxidative stress injury.
• Wnt proteins are cysteine-rich glycosylated proteins that can affect neuronal development, immunity, tissue fibrosis, angiogenesis, stem cell proliferation, and cancer. Wnt pathways that function in conjunction with circadian clock gene pathways, such as TIMELESS, may promote new angiogenesis and tumorigenesis. Furthermore, disruption of circadian rhythms with sleep fragmentation may increase the risk for developing cancer and other circadian genes that include *hClock* also may promote the metastasis of colorectal cancer through the enhanced expression of angiogenesis-related genes.

## Abbreviations:

AD: Alzheimer’s disease
DM: diabetes mellitus
EPO: erythropoietin
FoxOs: mammalian forkhead transcription factors
HD: Huntington’s disease
NCDs: non-communicable diseases
mTOR: the mechanistic target of rapamycin
mTORC1: mTOR Complex 1
mTORC2: mTOR Complex 2
PER2: period2
PRAS40: proline rich Akt substrate 40 kDa
SIRT1: the silent mating type information regulation 2 homolog 1 (*Saccharomyces cerevisiae*)
US: United States
USD: United States Dollars
Wnt: *wingless*
